# A high-resolution geospatial analysis of radiation therapy access in the Philippines

**DOI:** 10.3332/ecancer.2026.2113

**Published:** 2026-04-29

**Authors:** Juzzel Ian Zerrudo, Fjorda Kim Rubian-Zerrudo

**Affiliations:** 1 Medical Physics Section, Radiation Oncology Unit, Cancer Center, Batangas Medical Center, Batangas City 4200, Philippines; 2 Physics and Geology Unit, Department of Physical Sciences and Mathematics, College of Arts and Sciences, University of the Philippines Manila, Manila 1000, Philippines; 3 Radiology Department, Batangas Medical Center, Batangas City 4200, Philippines; a https://orcid.org/0009-0003-7332-7696

**Keywords:** radiotherapy, health services accessibility, geospatial analysis, health equity, Philippines, E2SFCA

## Abstract

**Background:**

In 2019, the Philippines enacted the National Integrated Cancer Control Act (NICCA) to decentralise cancer care. However, implementation remains limited by a lack of data to guide effective resource allocation. This study provides high-resolution, quantitative baseline data of radiotherapy (RT) accessibility in Luzon, the country’s largest island group, to identify priority areas for infrastructure development.

**Methods:**

A cross-sectional geospatial analysis for 698 municipalities was conducted using the Enhanced Two-Step Floating Catchment Area (E2SFCA) model, integrating provider capacity, population demand and calibrated travel time. Access was modelled under two scenarios: one including all 43 RT facilities and another restricted to the 12 public centres. To quantify financial toxicity, we developed a novel metric, the travel-cost-to-wage ratio. We performed spatial autocorrelation and multivariable regression analyses to identify geographic clusters and key determinants of access.

**Results:**

Profound inequities in RT access were identified. Considering all facilities, 240 municipalities (34.4%) home to 7.5 million people, had no potential access within a 120-minute catchment. The public-only scenario revealed a near-total system inadequacy, with 12.2 million people lacking access. Spatial analysis confirmed a stark core-periphery pattern, with a well-served core centred around the national capital and along the primary north-south expressway network, contrasted with vast, statistically significant ‘RT deserts’ in outlying regions. For 4.9 million people, a single round-trip to the nearest public facility costs more than a day’s minimum wage; this travel-cost-to-wage ratio was the strongest predictor of access (odds ratio = 0.83, p < 0.001).

**Conclusion:**

Spatial access to RT in Luzon is profoundly inequitable, characterised by a stark core-periphery pattern and an inadequate public system that leaves millions reliant on private care or entirely without access. This study provides a data-driven roadmap to guide the strategic implementation of NICCA, ensuring that future resource allocation targets the identified RT deserts to address these critical gaps in cancer care.

## Background

Current projections indicate that annual global cancer incidence will surpass 35 million by 2050, marking a 77% increase from 2022 levels [1]. This escalating burden falls disproportionately on low- and middle-income countries (LMICs), where health systems are often overextended, and resources are scarce [1–3]. Radiotherapy (RT), indicated for over half of all cancer patients, is an essential modality for cancer treatment and a critical component of any effective cancer control strategy [4, 5]. However, its high capital cost and the need for specialised infrastructure and personnel pose challenges in resource-limited settings [2, 6–9]. Despite accounting for over three-quarters of the world’s population, LMICs possess only a small fraction of the required treatment machines, leaving vast populations with no access to this vital care [7, 10–12]. This inequity manifests as a distinct geography of care, with services centralised in affluent urban centres, systematically marginalising rural and impoverished communities.

The Republic of the Philippines, an archipelagic LMIC in Southeast Asia, presents as a compelling illustration of this global challenge [13]. While cancer is a leading cause of mortality, the country’s RT landscape is characterised by a significant maldistribution of resources [14–17]. Over a third of all facilities are concentrated in the National Capital Region (NCR), which accounts for only 10% of the national population [15–18]. This centralisation forces patients from underserved regions to undertake long and costly journeys. This travel burden extends beyond time and distance, encompassing significant out-of-pocket expenditures for transport, accommodation, treatment and lost wages [15, 17, 18]. For many, this financial toxicity leads to treatment delays, interruptions or abandonment, directly contributing to poorer clinical outcomes [18, 19].

In response, the Philippine government enacted Republic Act 11215 or the National Integrated Cancer Control Act (NICCA) in 2019, which mandates the establishment of a network of regional cancer centres to decentralise services, improve access and make cancer care more equitable and affordable for all Filipinos [20]. However, this vital policy initiative is critically challenged by an evidence gap. While the strategic goal is clear, the lack of granular, quantitative data to identify the regions most in need of access is a significant limitation. To allocate finite resources for maximum impact and to address the deep-seated inequities in access, a detailed, data-driven understanding of the current accessibility landscape is needed.

This study was designed to directly address this policy need by employing a sophisticated geospatial analysis framework. Traditional metrics, such as simple provider-to-population ratios, are insufficient because they ignore the crucial spatial dimensions of access [3, 21]. Geospatial analyses have been instrumental in quantifying barriers and access, and in informing optimal locations for new RT facilities [3, 22–26]. However, most geospatial studies on RT access use travel time as the primary measure, which fails to account for the relationship between the capacity of existing centres and the potential demand [3, 18, 22–25]. In an LMIC setting with limited capacity and high demand, travel time alone does not fully capture the extent of the problem. Furthermore, existing studies do not differentiate between public and private RT services. This distinction is vital for understanding access for economically disadvantaged patients who cannot afford out-of-pocket costs.

Geospatial methods, particularly the Enhanced Two-Step Floating Catchment Area (E2SFCA) model, offer a more robust framework by integrating provider capacity, population demand and travel impedance with a distance-decay function that realistically models patient behaviour [21, 27]. Although this framework is widely applied in healthcare studies [21, 27–30], this work represents the first application of the E2SFCA model to RT access in the Philippines. Critically, this study is the first to apply a spatial capacity-demand model to disaggregate public from private services a vital distinction in a mixed health system where access and the ability to pay are significant barriers. The primary objectives of this study are: (1) to measure spatial accessibility to all RT centres in Luzon at the municipal level using the E2SFCA model, (2) to separately quantify access to public RT centres to assess the public system’s capacity and identify populations reliant on private care and (3) to identify key socioeconomic determinants of RT access.

## Methods

### Study design and area

This study was a cross-sectional, population-based geospatial analysis of RT accessibility. The geographical scope is confined to Luzon, the largest and most populous island group in the Philippines, encompassing 698 municipalities across the entirety of mainland Luzon. This includes the NCR and the surrounding regions of Central Luzon (Region III) and CALABARZON (Region IV-A), as well as the Ilocos Region (Region I), Cagayan Valley (Region II), Bicol Region (Region V) and Cordillera Administrative Region (CAR) [31]. While administratively part of Luzon, the island provinces of Mindoro, Marinduque, Romblon and Palawan were excluded to maintain a focus on the contiguous landmass where road-based travel is the primary mode of transport. This focused approach is justified because Luzon is home to over half the country’s population and the vast majority of its RT facilities, making it the centre of the national cancer care network and enabling the creation of a robust baseline for future nationwide studies [18, 31].

### Data sources

All datasets were aggregated from authoritative sources and integrated within a Geographic Information System (GIS) framework using QGIS (version 3.40 Bratislava LTR) [32]. Municipal-level administrative boundaries were obtained from the Philippine National Mapping and Resource Information Authority shapefile, retrieved from the Humanitarian Data Exchange [33]. The road network was sourced from OpenStreetMap (OSM) and was restricted explicitly to major road classifications (motorway, trunk, primary, secondary, tertiary) to model inter-municipal and inter-provincial travel [34]. Population origins were defined as the population-weighted centroids of each municipality and were derived from the WorldPop 2020 1-km high-resolution dataset [35]. This approach ensured that demand nodes are located at the geographic centre of the population within each municipality.

A comprehensive list of licensed RT centres and the number of linear accelerators (LINACs) per facility, current as of August 2025, was obtained from the Philippine Food and Drug Administration’s Center for Device Regulation, Radiation Health and Research. Each facility was geolocated using Google Maps (Google, Mountain View, California). In the absence of publicly available facility-level patient census data, annual capacity was modelled at 450 patients per LINAC, a figure consistent with recent global estimates [2, 7, 11].

Annual municipal cancer cases were estimated by applying the 2017 Philippine age-specific cancer incidence rates (the most recent public data for the Philippines) from the International Agency for Research on Cancer (IARC) [36] to 2020 age-stratified municipal population data from the Philippine Statistics Authority (PSA) [37]. To quantify municipal RT demand, the estimated number of cancer cases was multiplied by a RT utilisation rate of 64% [2, 3]. Socioeconomic variables included the 2021 municipal-level poverty incidence rate from the PSA [38] and the number of hospital beds per municipality from the Department of Health’s National Health Facility Registry [39].

### Travel network and travel time calibration

To ensure a realistic travel impedance model, a routable road network was created in QGIS using OSM data. Variable speed profiles were assigned to road segments based on municipal population as a proxy for traffic congestion, with baseline speeds derived from a national transportation planning report [40]. The travel times were calibrated against the results of Google Maps Distance Matrix API, which served as the reference standard. For each province, travel times were queried from every municipality to a central destination. OSM network speeds were then iteratively adjusted until the mean absolute error between the model’s travel times and the API baseline was under 25 minutes, and the mean absolute percentage error was below 20% for each region. The final calibrated model was used to generate a comprehensive origin-destination travel-time matrix using QGIS Network Analysis Toolbox (QNEAT3).

### The E2SFCA model

Potential spatial access was quantified using the E2SFCA model. This approach advances beyond simple provider-to-population ratios by accounting for facility capacity, local population demand and travel-time impedance [21, 28]. A 120-minute travel-time catchment threshold was selected, consistent with the literature identifying this duration as a critical barrier to adherence to daily RT schedules [3, 19, 25, 41]. Within this catchment, a continuous Gaussian function was used as a distance-decay weight to model the decreasing probability of facility utilisation with increasing travel time [28]. Before the analysis, all island municipalities without direct road access to the Luzon mainland were assigned a spatial access score of zero. For all remaining municipalities, spatial accessibility scores were calculated using the E2SFCA framework.

## The E2SFCA calculation proceeds in two steps for each municipality:

Step 1: Provider-to-population ratio. For each RT centre (j), a ratio (R_j_​) is calculated by dividing its capacity (S_j_​, i.e., total annual patient slots) by the sum of the potential RT demand (D_k_-​) located within its 120-minute catchment (t_0_​). Each population point within the catchment is weighted by a Gaussian distance-decay function G(t_kj_, t_0_), which gives greater weight to populations closer to the RT centre [28]. The ratio is expressed as follows:

$$R_{j}=\frac{S_{j}}{\Sigma_{k\in \left\{t_{\mathrm{kj}}\leq t_{0}\right\}}G\left(t_{\mathrm{kj}},t_{0}\right)D_{k}}$$

where the Gaussian distance-decay function is defined as follows:

$$G(t_{\mathrm{kj}},t_{0})={\{}_{0,t_{\mathrm{kj}}>t_{0}}^{\frac{e^{-(\frac{1}{2})\times (\frac{t_{\mathrm{kj}}}{t_{0}}{)}^{2}}-e^{-(\frac{1}{2})}}{1-e^{-(\frac{1}{2})}},t_{\mathrm{kj}}\leq t_{0}}$$

where t_kj_ represents the travel time between population origin k and RT centre j, and t_0_ is the 120-minute travel-time threshold. This step calculates a capacity-to-demand ratio for each treatment centre, accounting for the increased likelihood of nearby populations to utilise more adjacent RT centres.

Step 2: Accessibility score. For each municipal population centroid (k), the spatial accessibility score (Ak) is calculated by summing the ratios (R_j​_) of all RT centres within its 120-minute travel-time threshold (t_0_). These ratios are also weighted by the same Gaussian distance-decay function G(t_kj_, t_0_) to reflect the diminishing likelihood that patients travelling to more distant RT facilities. The accessibility score is expressed as follows:

$$A_{k}=\sum_{j\in \{t_{\mathrm{kj}}\leq t_{0}\}}G(t_{\mathrm{kj}},t_{0})R_{j}$$

This step calculates an overall access score for each municipality by summing the capacity ratios of all centres within its reach, prioritising centres with higher capacity-to-demand ratios and closer proximity to the population origin.

### Statistical analysis

To quantify the economic friction of travel, we developed a novel metric: the travel-cost-to-wage ratio. For each municipality, the round-trip cost to the nearest RT facility was estimated using the 2023 national fare matrices from the Land Transportation Franchising and Regulatory Board [42]. This calculation was based on jeepney fares for distances of 50 km or less and on bus fares for longer trips. The estimated round-trip cost was then divided by the official local daily minimum wage for that municipality, as stipulated by the respective Regional Tripartite Wages and Productivity Boards [43]. This quantity creates a standardised measure of financial burden relative to local earning capacity. It is important to note that this metric is only a conservative estimate of the actual economic burden of daily radiation treatment. This ratio accounts only for the direct transport fares using standard public transportation. It does not include other significant out-of-pocket expenses, such as food, lodging, lost productivity or the costs associated with an accompanying caregiver.

Two multivariable regression models were used to identify determinants of access. First, a binomial logistic regression model was used to predict the presence or absence of access. Second, an ordinary least squares (OLS) linear regression model was used to analyse the determinants of access levels among municipalities with non-zero scores. Independent variables for both models included municipal poverty incidence rates, total hospital beds per municipality, population density and the travel-cost-to-wage ratio. These variables were selected to capture key dimensions of the socioeconomic and healthcare landscape. Population density served as a proxy for urbanisation and demand concentration, which was hypothesised to be positively associated with access. Municipal poverty incidence rate represented the baseline socioeconomic vulnerability of a community and was expected to be inversely correlated with access. The number of hospital beds served as an indicator of the local healthcare infrastructure, testing whether RT access is primarily a function of overall health system development. Finally, the travel-cost-to-wage ratio was used to directly measure the economic friction imposed by geographic distance, isolating it from baseline poverty. All models were checked for multicollinearity using the Variance Inflation Factor.

Finally, to identify statistically significant geographic patterns, a spatial autocorrelation analysis was performed using the Local Moran’s I statistic. This technique identifies spatial clusters of high access (High-High hotspots), low access (Low-Low coldspots) and spatial outliers, providing statistical validation for the observed geographic patterns. Statistically significant geographic clusters of low spatial access, identified via Local Moran’s I analysis, are hereafter referred to as ‘RT deserts’.

## Results

### Landscape of RT access in Luzon

**Figure 1.**
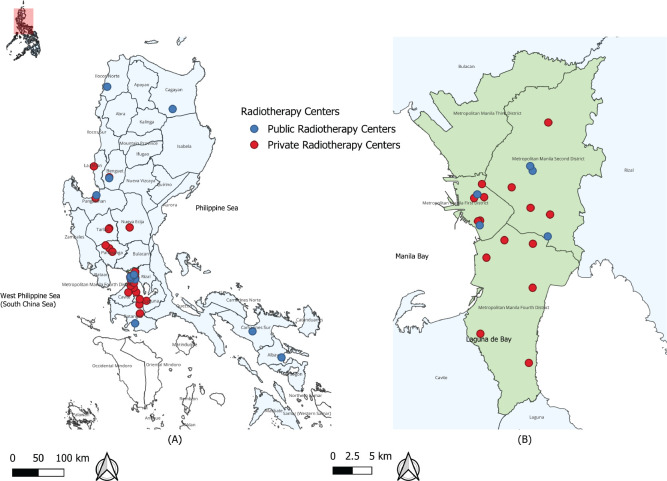
Distribution of Public and Private RT Centres in Luzon, Philippines. The map displays the locations of the 43 RT centres across the study area of Luzon (a), with the highly concentrated NCR (b). The 12 public facilities are marked with blue circles and the 31 private facilities with red circles, illustrating the heavy clustering of services within and around the metropolitan core.

**Figure 2.**
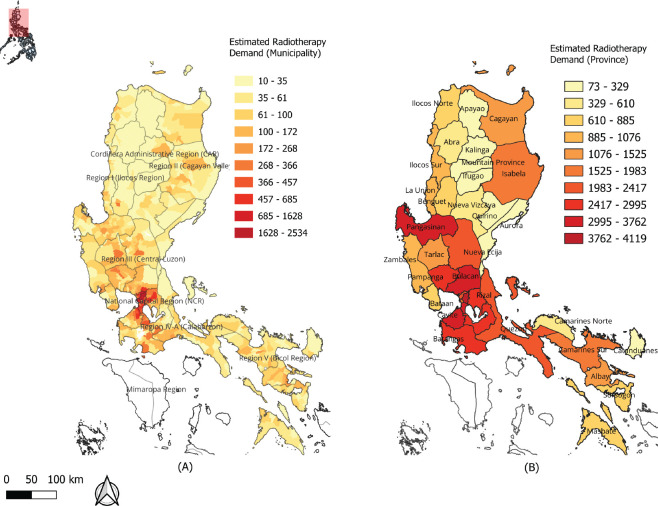
Estimated annual RT demand in Luzon. The maps illustrate the estimated annual number of new cancer patients requiring RT at the (a): municipal level and (b): provincial level. Darker shades of red indicate a higher concentration of demand. The highest demand is concentrated in the densely populated municipalities of the NCR, CALABARZON (Region IV-A) and Central Luzon (Region III).

As of August 2025, the island of Luzon hosted 43 RT centres equipped with a total of 54 licensed LINACs (Figure 1). The distribution is heavily skewed, with 20 centres (46.5%) and 29 LINACs (54%) located within the NCR. The public sector was limited, comprising only 12 centres, five of which were situated in the NCR. The total estimated annual RT demand for the study area was 56,605 patients, with the highest demand concentrated in the populous regions of CALABARZON, Central Luzon and the NCR (Figure 2).

**Figure 3.**
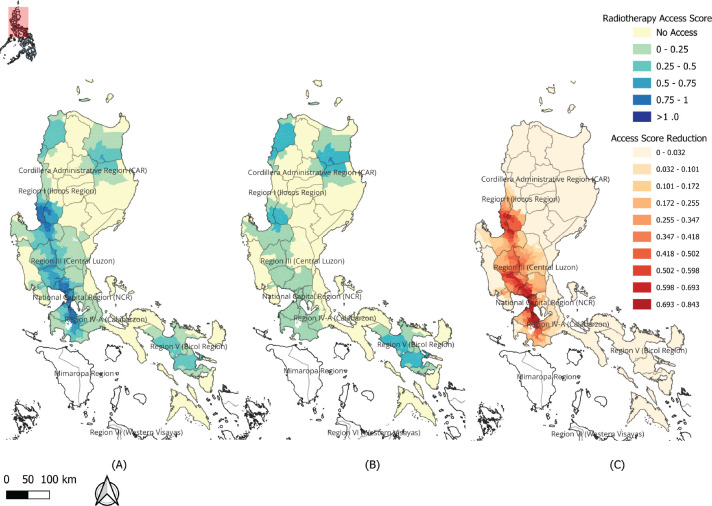
Spatial Accessibility to RT in Luzon using the E2SFCA Model. Municipal-level potential spatial access scores are shown for (a) all 43 public and private RT centres and (b) the 12 public-only centres. The E2SFCA score is a measure of provider capacity relative to potential population demand within a 120-minute catchment, with higher scores (darker blue) indicating better access and lower scores (yellow to light blue) indicating poorer access. Map (c) shows the absolute decrease in the access score when private facilities were excluded, directly highlighting the municipalities most reliant on the private sector for care.

The E2SFCA analysis, incorporating all 43 public and private centres, revealed significant deficits in potential spatial access. As detailed in Table 1, a total of 240 of 698 municipalities (34.4%) had a spatial access score of zero, indicating a complete lack of access within 120 minutes of travel. This geographic gap affected over 7.5 million people, representing 12.9% of Luzon’s population and an estimated 15.0% of the annual RT demand (Table 3). Furthermore, 183 municipalities (26.2%), home to 11.3 million people, fell into the ‘Least Access’ category (score > 0–0.25), where potential demand outstrips supply by a factor of four or more. Cumulatively, over 60% of municipalities, serving more than 18 million people, have either no or very lowpotential access to RT. As shown in Figure 3a, this disparity exhibited a core-periphery pattern: the ‘core’ of high access included the NCR and the linear development corridor along Luzon’s primary north-south expressway network, while leaving the municipalities outside this corridor as a vast, underserved periphery.

When private facilities were excluded, the analysis revealed the profound inadequacy of the public RT system. The number of municipalities with zero access increased from 240 (34.4%) to 320 (45.9%), and the affected population nearly doubled, from 7.5 to 12.2 million, accounting for over one-fifth of Luzon’s population (Table 2). In terms of clinical burden, this equates to 13,304 (23.5% of the total demand) estimated cancer patients requiring RT residing in areas with no public RT access (Table 3).

Furthermore, the quality of access deteriorated significantly; only a single municipality achieved a ‘Moderate Access’ score in the public-only scenario, with none reaching ‘Good’ or ‘Excellent’ levels. The vast majority (70.6%) of the population or 37,786 cancer patients with theoretical access to a public facility, fell into the ‘Least Access’ category (see Tables 2 and 3). This indicates that even where public services technically exist, the potential demand significantly overwhelms the capacity, suggesting severe congestion and long wait times. This finding demonstrates a heavy systemic reliance on the private sector for RT services in Luzon. This makes RT access effectively contingent on a patient’s ability to pay out-of-pocket, as national health insurance (PhilHealth) premiums were often insufficient to cover the total treatment costs in private facilities [17, 18].

### Statistical identification of RT deserts

**Figure 4.**
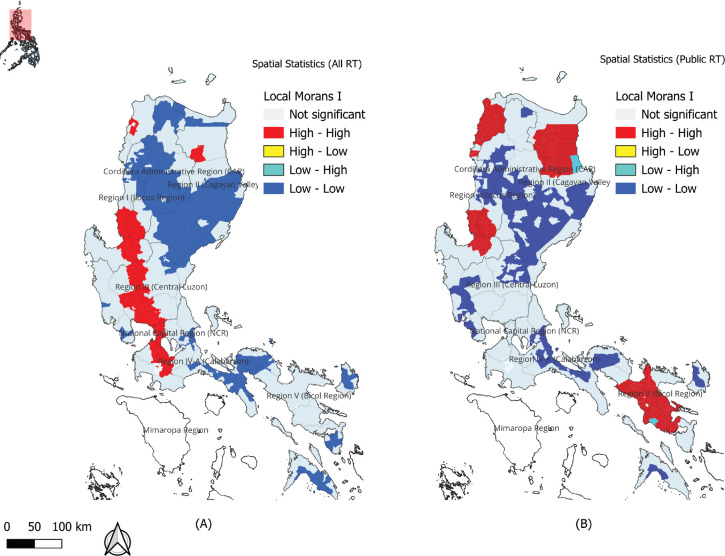
Statistically Significant Spatial Clusters of RT Access. The maps display the results of the Local Moran’s I spatial autocorrelation analysis for (a) all centres and (b) public-only centres. This analysis identifies statistically significant geographic clusters, including hotspots of high access (High-High, red) and cold spots of low access (Low-Low, blue). In this study, the Low-Low clusters are formally defined as ‘RT deserts’.

Spatial autocorrelation analysis provided statistical validation for these observed patterns. The Local Moran’s I statistic identified a large, contiguous ‘High-High’ cluster (hotspot) of RT access encompassing the NCR and extending into the surrounding highly urbanised provinces along the primary north-south expressway network (Figure 4a). In stark contrast, the analysis identified extensive, statistically significant ‘Low-Low’ clusters or cold spots, which were formally designated as ‘RT deserts’ (Figure 4b). These included vast territories in Cagayan Valley, the entirety of the CAR, large parts of the Bicol Region, eastern portions of Laguna and Rizal provinces and the province of Quezon. This analysis moved beyond simple visualisation to provide a statistically validated map of priority areas for intervention.

### The dual burden of travel time and financial toxicity

**Figure 5.**
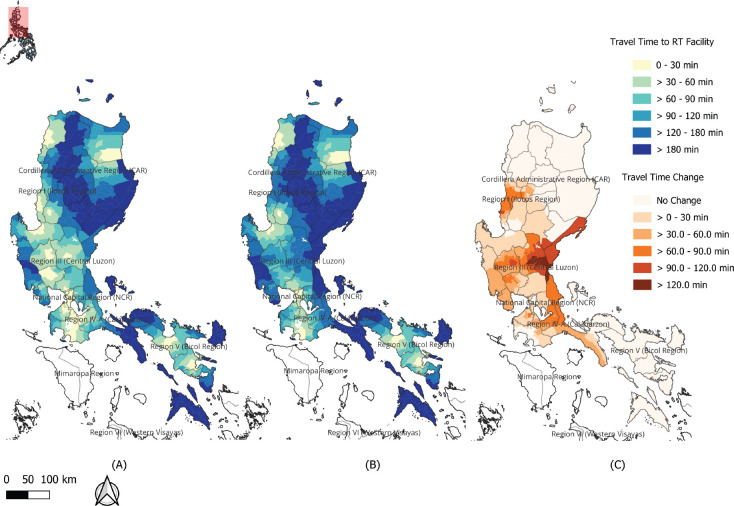
One-Way Travel Time to the Nearest RT Centre. The maps display the calibrated one-way travel time in minutes from each municipal centroid to (a) the nearest of any of the 43 RT centres and (b) the nearest of the 12 public RT centres. Darker shades indicate longer travel times. Map (c) visualises the additional travel time incurred when relying solely on the public system.

Distance imposed a dual penalty: excessive travel time and catastrophic financial costs. While two-thirds of the population lived within a 60-minute drive of any RT centre (Figure 5a; Table 4), this figure plummeted to just under half (49.6%) when considering public centres only (Figure 5b; Table 4). Notably, nearly 5 million people (8.5%) lived more than 3 hours fro≠≠m the nearest public facility, a prohibitive distance for daily treatment regimens.

**Figure 6.**
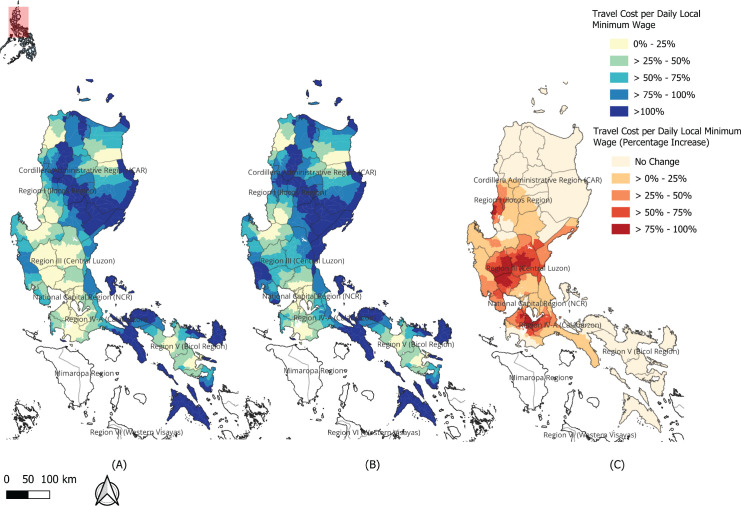
Economic Burden of Travel to RT Centres. The maps illustrate the financial toxicity of travel, measured as the estimated round-trip cost to the nearest facility as a percentage of the local daily minimum wage (travel-cost-to-wage ratio). The analysis is shown for (a) any RT centre and (b) public-only RT centres, with darker shades representing a higher travel-cost-to-wage ratio. Map (c) visualises the increase in the travel-cost-to-wage ratio when relying solely on public facilities.

The travel-cost-to-wage ratio quantified the economic impact of this travel (Figure 6 and Table 5). For access to the nearest public facility, over 50% of the population lived in municipalities where a single round-trip costs more than 25% of the local daily minimum wage. Most critically, for 159 towns, home to nearly 4.9 million people, a single round-trip journey to the nearest public RT centre was estimated to cost more than a full day’s wages. This level of financial burden transforms a geographic barrier into an effectively insurmountable economic one, rendering treatment practically inaccessible for the working poor.

### Determinants of RT access

The multivariable regression models identified the statistical drivers of the observed access disparities, confirming that the economic barriers associated with geographic distance were a stronger predictor of access than a municipality’s baseline poverty level alone. The logistic regression model, which predicted the presence or absence of any potential RT access, found that the travel-cost-to-wage ratio was the strongest and the only statistically significant predictor. For every one percentage-point increase in the travel cost as a percentage of the local daily wage, the odds of a municipality having any potential access to RT decrease by 17% (odds ratio = 0.83, *p* < 0.001). These results provide robust statistical evidence that the economic burden of travel is a primary factor in determining whether a community has potential spatial access to a RT centre.

The OLSs regression model analysed the factors determining the magnitude of access among the 458 municipalities with non-zero scores. The results, presented in Table 6, showed that higher population density was associated with greater access, reflecting the concentration of facilities in urban centres. Concurrently, a higher travel-cost-to-wage ratio was significantly associated with lower accessibility. Notably, once the travel cost variable was included in the model, a municipality’s poverty incidence ceased to be a significant predictor. This suggests that the mechanism of inequity is not simply poverty, but rather the direct economic and logistical burden imposed by geography.

## Discussion

This study provided the first high-resolution, methodologically robust quantification of potential spatial access to RT in the Philippines. The principal findings revealed a landscape of significant inequity characterised by a distinct core-periphery pattern, where adequate access was concentrated in the national capital and along the primary north-south expressway network, leaving vast, contiguous RT deserts in outlying regions. Over 7.5 million people resided in areas with no potential access to any RT facility within a 2-hour travel time. The analysis further exposed the near-total inadequacy of the public RT system, which, on its own, fails to provide access to over 12 million people. This results in a heavy reliance on the private sector, effectively making a patient’s ability to pay the primary determinant of access. These findings quantify the significant challenge this poses to the Philippine government’s cancer control efforts.

These findings both corroborate and substantially expand upon previous studies of the Philippine RT landscape [15, 16, 18]. While the work of Flores *et al* [15] described the landscape of barriers and Eala *et al* [18] quantified the travel burden in terms of distance, time and cost, this study advances this understanding in three critical ways. First, by employing the E2SFCA model, this work moves beyond simple proximity to a framework that accounts for both facility capacity and population demand. This work revealed that even geographically proximate centres may be functionally inaccessible because potential demand exceeds the facility’s capacity. Second, the novel travel-cost-to-wage ratio reframes the economic barrier from a simple out-of-pocket cost to a direct measure of financial toxicity relative to earning capacity, providing a more nuanced understanding of the economic friction that drives cancer care inequity. Third, the formal, statistical identification of RT deserts using spatial autocorrelation offers an objective, evidence-based framework for prioritising areas for intervention, a significant advance over descriptive mapping.

The identification of a well-served linear development corridor that follows Luzon’s primary north-south expressway network reframes the problem of access not exclusively as a challenge of health system planning, but as a direct consequence of national development patterns. The literature supports the finding that transportation infrastructure is a primary indicator of healthcare access [44, 45]. This is also directly applicable to RT, where a greater distance to a treatment centre is statistically associated with higher cancer mortality and acts as a prohibitive barrier to treatment [46]. The concentration of services along this corridor can be explained by research from rural China, which found that major transportation infrastructure expansion tends to be most beneficial to areas with already better spatial access [44]. This system creates a self-perpetuating feedback loop in which well-connected areas attract further investment and services, reinforcing the centralisation of the healthcare system and marginalising less-developed regions.

This dynamic of centralisation directly supports our regression finding that economic friction – as measured by the travel-cost-to-wage ratio – is a significant predictor of poor access. This conclusion is supported by studies showing that travel burden is directly associated with decreased RT utilisation and poorer oncologic outcomes [41, 46]. These findings confirm that geographic isolation and inadequate road networks are major drivers of health inequity, a phenomenon particularly acute in topographically challenging areas. For instance, Tanou *et al* [47] found in Benin that the adverse effects of distance on healthcare use were most pronounced in the country’s mountainous, rural northern region, where road infrastructure was less developed. This provides a direct international parallel to the RT deserts identified in the mountainous Cordillera Region and the geographically vast Cagayan Valley and Quezon province, underscoring the need for a deliberate break from historical development patterns that mandate that health equity, not just economic convenience, guide future infrastructure investment.

The situation in Luzon also serves as a representative case study of the challenges confronting many LMICs, where severe equipment shortages and over-reliance on centralised urban facilities are well-documented problems [7, 11]. A key contribution of this study is the identification of the primary mechanism driving these inequities. The regression analyses demonstrated that the travel-cost-to-wage-ratio – a novel metric of economic friction – was a more powerful predictor of access than municipal-level poverty incidence. This indicated that geographic isolation itself, and the direct financial toxicity of overcoming it, is the most significant barrier to cancer care. While poverty and geographic isolation are often correlated, this finding offers a crucial policy insight: to be effective, interventions must be geographically targeted to reduce travel burdens and overcome economic barriers. Financial aid alone may prove insufficient for patients living hundreds of kilometres from the nearest facility. This concept of economic friction due to distance is a critical consideration for health systems planning in geographically challenged settings.

The policy implications of these findings are direct and urgent, particularly for the implementation of the NICCA. While the law’s mandate to establish a network of regional cancer centres represents a significant step towards health equity, its success, however, hinges on the strategic placement of these new facilities. This study provides the evidence-based roadmap for such planning. The spatial accessibility maps and, most importantly, the statistically validated ‘Low-Low’ clusters (Figure 4) provide a data-driven framework for identifying where new public RT centres are urgently needed. Prioritising new facility investments in the identified RT deserts – specifically in Cagayan Valley, the CAR, Zambales, Rizal, Quezon and the Bicol Peninsula – would yield the most significant impact in reducing travel burdens and improving access for underserved communities.

Furthermore, our findings provide a powerful rationale for strengthening patient support systems to mitigate the financial toxicity of travel. As building new infrastructure is a long-term solution, immediate measures are needed to address the economic burden of travelling to the RT facility. The Cancer Assistance Fund, established under NICCA, should be leveraged to expand programs that provide subsidies for patient and caregiver transportation and accommodation [20]. This is a critical stopgap measure to prevent treatment abandonment due to indirect costs. Finally, the stark contrast between spatial accessibility under a total versus public-only system is a clear call for sustained investment in government health infrastructure. Achieving the universal health coverage goals of NICCA and the Universal Health Care Law (RA 11223) [48] will remain impossible without a deliberate, long-term strategy to build public oncology capacity outside of the NCR. This includes not only investing in equipment and infrastructure but also in recruitment and retention strategies to attract, train and maintain oncologists, medical physicists and other specialised personnel in provincial areas [17–20].

Finally, the methodological framework developed in this study offers a transferable framework for other LMICs seeking to generate actionable evidence for equitable health resource allocation. This three-part framework is broadly applicable to any resource-constrained setting with a mixed public-private health system. The components include: (1) the application of a robust supply-and-demand model, such as the E2SFCA, to move beyond simple proximity measures; (2) the critical disaggregation of public versus private services to assess the functional capacity of the public system and quantify reliance on out-of-pocket care and (3) the use of a standardised, intuitive metric like the travel-cost-to-wage ratio to measure the financial burden of geographic barriers. This comprehensive approach serves as a powerful model for leveraging geospatial science to produce a clear, data-driven roadmap for health infrastructure planning and policy reform worldwide.

### Strengths and limitations

This study has several notable strengths. First, it is the first to apply the robust E2SFCA framework to RT access in the Philippines, providing a more sophisticated measure of potential access than previously available. Second, the novel disaggregation of the public and private sectors offers a critical, real-world assessment of the public system’s capacity and quantifies the population’s reliance on private care. Third, the development and application of the travel-cost-to-wage ratio introduces a standardised metric for measuring the economic friction of travel, a key barrier in LMICs. Fourth, the high-resolution, municipal-level analysis yields granular, actionable data directly applicable to sub-national health planning. Finally, the use of spatial autocorrelation to statistically identify RT deserts provides a rigorous, objective basis for policy decisions.

Nevertheless, the study’s limitations must be acknowledged. First, the analysis is subject to the ecological fallacy because it is conducted at the municipal level, and, consequently, access experiences may vary across individuals within a municipality. Second, the E2SFCA model measures potential spatial access, rather than actual utilisation. The latter is influenced by multiple non-geographic factors such as physician referral patterns, patient health literacy, cultural beliefs, treatment costs and the perceived quality of care, which were outside the scope of this analysis. Third, the available input data also constrains the analysis; it relies on the 2017 cancer incidence rates (the latest publicly available data for the Philippines from IARC) and uses a standardised benchmark for LINAC capacity, which may not capture the operational variability of individual centres. Lastly, the models for both travel time and cost are conservative estimates. The travel time model cannot account for all real-world complexities, such as unpredictable traffic or weather, and the travel-cost-to-wage ratio does not include high indirect costs such as lost wages, food, lodging or expenses for an accompanying caregiver.

### Future research

This study lays the groundwork for several critical avenues of future research. An immediate priority is to expand this analysis to the Visayas and Mindanao regions, which will require developing more complex, multi-modal travel network models to accurately capture inter-island travel. Second, the quantitative findings of this study should be complemented by qualitative research. In-depth interviews and focus group discussions with patients, caregivers and healthcare providers within the identified RT deserts would provide invaluable insights into the lived experiences of access barriers and help shape more culturally and contextually appropriate interventions. Finally, the analytical framework established in this study extends beyond descriptive analysis to serve as a powerful predictive tool for policy planning. By modelling the placement of a new public RT centre in a RT desert such as Cagayan Valley, the framework can quantify the projected increase in the E2SFCA score for surrounding municipalities and the reduction in the population facing prohibitive travel burdens. Such scenario modelling would allow the Department of Health to compare the impacts of different investment strategies and optimise the placement of new facilities to maximise impact and ensure the goals of the NICCA and UHC laws are met efficiently.

## Conclusion

Spatial access to RT in Luzon, Philippines, is profoundly inequitable, characterised by a distinct core-periphery pattern that leaves millions without potential access, particularly in the island’s northern and southeastern regions. The public RT system is critically inadequate, requiring a heavy reliance on private services and rendering financial capacity a primary determinant of access. This study demonstrated that the economic friction of geographic isolation, quantified by the travel-cost-to-wage ratio, is a primary driver of this disparity. The findings presented here provide a critical, data-driven tool for the Philippine government and a clear roadmap for the strategic implementation of the NICCA. By targeting new resources to the statistically identified RT deserts, policymakers can systematically reduce these geographic barriers and build a more equitable cancer care system for all Filipinos.

## Conflicts of interest

The authors are employed by Batangas Medical Center and declare no other competing interests.

## Funding

This research received no funding.

## Author contributions

Juzzel Ian Zerrudo: Conceptualisation; Data curation; Formal analysis; Investigation; Methodology; Project administration; Visualisation; Writing – original draft; Writing – review & editing. Fjorda Kim Zerrudo: Formal analysis; Visualisation; Writing – review & editing.

## Figures and Tables

**Table 1.** Summary of spatial accessibility scores of municipalities in Luzon to RT centres within 120 minutes, by facility type

Access category	Access score range	All RT centres (n = 43)	Public RT centres (n = 12)
		**Municipalities,** *n*** (%)**	**Municipalities,** *n*** (%)**
No access	0	240 (34.4%)	320 (45.9%)
Least access	>0–0.25	183 (26.2%)	286 (41.0%)
Low access	>0.25–0.50	153 (22.0%)	91 (13.0%)
Moderate access	>0.50–0.75	66 (9.5%)	1 (0.1%)
Good access	>0.75–1.0	54 (7.7%)	0 (0.0%)
Excellent access	>1.0	2 (0.3%)	0 (0.0%)

**Table 2.** Comparison of population spatial accessibility scores: all facilities versus public facilities only.

Access category	Access score range	All RT centres (n = 43)	Public RT centres (n = 12)
		**Population, *n* (%)**	**Population, *n* (%)**
No access	0	7,581,242 (12.9%)	12,219,763 (20.7%)
Least access	>0–0.25	11,256,282 (19.1%)	41,600,752 (70.6%)
Low access	>0.25–0.50	13,291,004 (22.5%)	4,981,535 (8.5%)
Moderate access	>0.50–0.75	12,183,999 (20.7%)	166,334 (0.3%)
Good access	>0.75–1.0	13,671,756 (23.2%)	0 (0.0%)
Excellent access	>1.0	984,101 (1.7%)	0 (0.0%)

**Table 3.** Estimated RT demand by access category: all facilities versus public facilities only.

Access category	Access score range	All RT centres (n=43)	Public RT centres (n = 12)
		**RT Demand,** *n*** (%)**	**RT Demand,** *n*** (%)**
No access	0	8491 (15.0%)	13304 (23.50%)
Least access	>0–0.25	11309 (19.98%)	37786 (66.75%)
Low access	>0.25–0.50	13028 (23.02%)	5343 (9.44%)
Moderate access	>0.50–0.75	11128 (19.66%)	172 (0.3%)
Good access	>0.75–1.0	11826 (20.89%)	0 (0.0%)
Excellent access	>1.0	823 (1.45%)	0 (0.0%)

**Table 4.** Population distribution of one-way travel time to nearest RT centre.

Travel time (minutes)	All RT centres	Public RT centres
	**Population, *n* (%)**	**Population, *n* (%)**
≤60 minutes	39,228,851 (66.5%)	29,252,655 (49.6%)
>60−90 minutes	8,681,947 (14.7%)	10,697,038 (18.1%)
>90−120 minutes	3,476,344 (5.9%)	6,798,928 (11.5%)
>120−180 minutes	3,588,123 (6.1%)	7,232,672 (12.3%)
>180	3,993,119 (6.8%)	4,987,091 (8.5%)

**Table 5.** Economic burden of travel to nearest RT centre.

Round-trip cost (% of daily minimum wage)	All RT centres	Public RT centres
	**Population, *n* (%)**	**Population, *n* (%)**
0%−25%	40,796,607 (69.2%)	29,283,020 (49.7%)
26%−50%	7,984,931 (13.5%)	11,794,253 (20.0%)
51%−75%	3,417,025 (5.8%)	8,866,301 (15.0%)
76%−100%	2,781,698 (4.7%)	4,157,182 (7.1%)
>100%	3,988,123 (6.8%)	4,867,628 (8.3%)

**Table 6.** Determinants of RT access: ordinary least squares regression and multivariable logistic regression models results

Variable	Logistic regression	Ordinary least squares regression
	**Odds ratio (95% CI), *p*-value**	**Coefficient (95% CI), *p*-value**
Population density (per km²)	1.001 (0.998–1.003), *p* = 0.610	1.46x10⁻⁵ (1.05x10⁻⁵, 1.87x10⁻⁵), *p* < 0.001
Number of hospital beds (*n*)	0.999 (0.991–1.007), *p* = 0.795	−2.55x10⁻⁵ (−5.83x10⁻⁵, 7.24x10⁻⁶), *p* = 0.127
Poverty Incidence (%)	1.015 (0.96–1.073), *p* = 0.608	−0.00152 (−0.00356, 5.33 x 10⁻⁴), *p* = 0.147
Travel-cost-to-wage-ratio (%)	0.829 (0.790–0.868), *p* < 0.001	−0.00783 (−0.00886, −0.0068), *p* < 0.001
Model fit	McFadden’s *R*² = 0.867	*R*² = 0.506
